# The Pandemic Influenza A (H1N1) 2009 Vaccine Does Not Increase the Mortality Rate of Idiopathic Interstitial Pneumonia: A Matched Case-Control Study

**DOI:** 10.1371/journal.pone.0088927

**Published:** 2014-02-25

**Authors:** Hiroshi Yokomichi, Shintaro Kurihara, Tetsuji Yokoyama, Eisuke Inoue, Keiko Tanaka-Taya, Shigeru Kono, Zentaro Yamagata

**Affiliations:** 1 Department of Health Sciences, Interdisciplinary Graduate School of Medicine and Engineering, University of Yamanashi, Chuo City, Yamanashi, Japan; 2 Infection Control and Education Center, Nagasaki University, Nagasaki City, Nagasaki, Japan; 3 Department of Health Promotion, National Institute of Public Health, Wako City, Saitama, Japan; 4 Department of Clinical Medicine (Biostatistics), School of Pharmacy, Kitasato University, Minato Ward, Tokyo, Japan; 5 Infectious Disease Surveillance Center, National Institute of Infectious Diseases, Shinjuku Ward, Tokyo, Japan; 6 Second Department of Internal Medicine, Nagasaki University School of Medicine, Nagasaki City, Nagasaki, Japan; The University of Tokyo, Japan

## Abstract

**Background:**

Evidence regarding the mortality rate after administration of the pandemic influenza A (H1N1) 2009 vaccine on patients with underlying diseases is currently scarce. We conducted a case-control study in Japan to compare the mortality rates of patients with idiopathic interstitial pneumonia after the vaccines were administered and were not administered.

**Methods:**

Between October 2009 and March 2010, we collected clinical records in Japan and conducted a 1∶1 matched case-control study. Patients with idiopathic interstitial pneumonia who died during this period were considered case patients, and those who survived were considered control patients. We determined and compared the proportion of each group that received the pandemic influenza A (H1N1) 2009 vaccine and estimated the odds ratio. Finally, we conducted simulations that compensated for the shortcomings of the study associated with adjusted severity of idiopathic interstitial pneumonia.

**Results:**

The case and control groups each comprised of 75 patients with idiopathic interstitial pneumonia. The proportion of patients who received the pandemic influenza A (H1N1) 2009 vaccine was 30.7% and 38.7% for the case and control groups, respectively. During that winter, the crude conditional odds ratio of mortality was 0.63 (95% confidence interval, 0.25–1.47) and the adjusted conditional odds ratio was 1.18 (95% confidence interval, 0.33–4.49); neither was significant. The simulation study showed more accurate conditional odds ratios of 0.63–0.71.

**Conclusions:**

In our study, we detected no evidence that the influenza A (H1N1) 2009 vaccine increased the mortality rate of patients with idiopathic interstitial pneumonia. The results, however, are limited by the small sample size and low statistical power. A larger-scale study is required.

## Introduction

After its initial identification in Mexico and the United States in April 2009, the novel influenza A (H1N1) 2009 virus spread worldwide. In response, the World Health Organization raised the pandemic alert level to phase 6 [1]. Seasonal influenza vaccines were not expected to protect against the influenza A (H1N1) 2009 virus [2]; therefore, the development of an efficient and safe pandemic influenza vaccine became an urgent need [3].

The suspected adverse events of seasonal influenza vaccines include acute disseminated encephalomyelitis and Guillain-Barré syndrome [4]–[8]. After the administration of 82.4 million doses of the influenza A (H1N1) 2009 vaccine, 48 case fatalities were noted by the U.S. Vaccine Adverse Event Reporting System [7]. Pandemic influenza A (H1N1) 2009 reached Japan in September 2009, and pandemic vaccines were administered to the Japanese population. Among patients with underlying disease, 131 died after receiving the vaccine, including 22 patients with chronic kidney disease, 12 with chronic obstructive pulmonary disease, and 11 with interstitial pneumonia [9]. Adverse events resulting from vaccination were not confirmed as the cause of death, and it was not confirmed that the vaccine exacerbated the underlying disease over the short or long term [10].

Idiopathic interstitial pneumonia (IIP) is a rare disease [11], [12] that is generally diagnosed by a respiratory physician based on the criteria published by the Japanese Respiratory Society. Patients with IIP may represent a high-risk group for post-vaccination mortality [7], [8]. The World Health Organization recommended that patients with underlying disease or those who are immunosuppressed receive priority for influenza A (H1N1) 2009 vaccination [13]. Patients with IIP are usually treated with systemic corticosteroids [11], [12], and the safety of seasonal influenza vaccines in patients with underlying diseases who are treated with systemic corticosteroids is controversial. To our knowledge, no studies have thus far tested if the mortality rate increased in patients with IIP after receiving the influenza A (H1N1) 2009 vaccine.

To clarify the risk of mortality associated with the influenza A (H1N1) 2009 vaccine, we conducted a matched case-control study of patients with IIP who died (case patients) and those who survived (control patients). The estimated prevalence of IIP is 13–20 of every 100,000 individuals [14]. Throughout their lives, patients with incurable IIP usually consult with respiratory physicians in Japanese hospitals registered with the Japanese Respiratory Society. We aimed to explore potential increases in mortality rate attributable to this vaccine in patients with IIP during the winter of 2009–2010 if they existed.

## Methods

To obtain the data in this case-control study, we sent surveys to pulmonary physicians in hospitals registered with the Japanese Respiratory Society. The physicians consulted clinical records to provide the requested information regarding their patients with IIP and then returned the surveys to our institution. After we obtained the completed surveys, we analyzed the matched case-control data.

### Case Definition and Control Selection

Patient case criteria included ≥18 years of age, a previous diagnosis of IIP, and death during the study period (October 1, 2009, to March 31, 2010) regardless of the cause. In this matched case-control study, we selected 1 IIP control (a patient who survived) for each IIP case (a patient who died) from the same hospital on the same day that the case patient died (index date). Simultaneously, the patient with IIP in the case-control pair was matched according to gender, age (±3 years), and follow-up period (years). The severity of IIP has been shown to increase gradually throughout the follow-up period; the median survival period of patients with IIP is 3–7 years after diagnosis [11], [14]. We ensured that the risk of death of the control patients was similar to that of the case patients. We used the follow-up period as an available standard to determine the severity of IIP, and we matched the case and control pairs on the index date [15]. When more than 1 control patient matched a case patient in the same hospital on the index date, the pulmonary physician selected the control patient according to alphabetical order. The on-site pulmonary physicians matched the case patient to the control patient. Thus, all information needed to determine a case-control pair was collected from clinical records on the index date. Moreover, we excluded control data that were obtained after the index date.

### Exposure to Vaccine

The vaccinated patients with IIP received a single subcutaneous dose of influenza A (H1N1) 2009 vaccine (0.5 mL), which consisted of purified, monovalent, non-adjuvanted, inactivated, split virions that contained 15 µg hemagglutinin specific for influenza A/California/7/2009 (H1N1). The vaccines were produced by the Kitasato Institute, the Chemo-Sero-Therapeutic Research Institute, DENKA SEIKEN Co. Ltd., or the Research Institute for Microbial Diseases of Osaka University; these were the only available vaccines that targeted the pandemic influenza virus. All of these vaccine preparations were of commercial grade and were licensed by the Ministry of Health, Labour and Welfare in Japan. Data of patients who received a single dose of the influenza A (H1N1) 2009 vaccine before the index date were reported by the physicians based on the clinical records.

### Covariates

Data on several confounding covariates were gathered simultaneously from clinical records. The covariates we identified included reasons for non-vaccination, seasonal influenza vaccination records, and history of cancer and diabetes mellitus. On-site pulmonary physicians assessed the physical conditions of the unvaccinated patients, referring to Fletcher-Hugh-Jones classification, measurement of arterial blood gases, images (X-ray, CT), and treatment itself. The physical condition of the patients was an additional question asked to the pulmonary physicians, and it was a reason that some patients were not vaccinated.

### Statistical Analysis

We determined associations between influenza A (H1N1) 2009 vaccination and the mortality rate by estimating the crude conditional odds ratio (OR) and its 95% confidence interval (CI). We used conditional logistic regression [16] to estimate the ORs and CIs conditioned by age, gender, and follow-up period. In addition, we simultaneously adjusted the analysis for confounders, which included comorbidity (i.e., diabetes mellitus and history of cancer), history of seasonal influenza vaccination, and reasons for non-vaccination (i.e., poor physical condition and shortage of vaccine), and we expected greater accuracy of the estimated ORs after this adjustment. For small-sample studies, we found that the exact permutation test yielded more accurate ORs and CIs than those generated by conventional conditional logistic regression methods; thus, we used the exact permutation test. We applied 95% CIs to our analyses instead of 2-sided p values because 95% CIs provide more information. In addition, we conducted 2 sensitivity analyses with (1) restriction of the primary outcome to death caused by exacerbation of IIP or pneumonia (70 pairs) and not to death from all causes and (2) restriction of the case patients to those not in poor physical condition (55 pairs).

### Simulation Study

A limitation of our study was the use of poor physical condition as a covariate. Data were gathered for this covariate to support a reason for non-vaccination in this study; however, we did not collect this information from vaccinated patients. The OR was adjusted for physical condition, which was always assigned not to be poor in vaccinated patients. This assignment would create positive bias for the OR. To estimate the less biased OR and extent of positive bias when adjusted for poor physical condition, we conducted a simulation study; we estimated a more genuine OR based on the assumption that all of the study patients’ physicians reported the physical condition of each patient. (a) The simulation condition was that using conditional logistic regression, a conditional OR was calculated for each of the potential probability values (0.1, 0.2, 0.3, 0.4, and 0.5) that were assigned to vaccinated patients for being in poor physical condition. This process is identical to the multiple-imputation method when it is used for binary missing data related to physical condition [17]; the covariate of whether a vaccinated patient was in poor physical condition or not is missing here. In 1 simulated data set, each vaccinated patient was assigned with the probability of either being or not being in poor physical condition. Each simulation calculates one conditional OR. For each of the 5 probabilities, the simulation was performed 10,000 times. The means of the adjusted conditional ORs and standard error were calculated based on 10,000 simulations for each probability. (b) The physical condition of patients may have been correlated with severity (follow-up period). Although no significant correlation between physical condition and follow-up period was detected in the unvaccinated patients in our data (OR = 1.14 per year, CI: 0.97–1.33), we assigned their binary physical condition to the vaccinated patients according to the coefficients of the logistic regression, and estimated the conditional OR. This simulation was also performed 10,000 times. Thereafter, we provided the mean and the standard error of OR in simulation (b).

All statistical analyses were performed using SAS version 9.3 (Cary, NC, USA).

### Ethics

The study protocol was approved by the Ethics Review Committee of the Faculty of Medicine at the University of Yamanashi. Approval was provided based on the ethical guidelines and the Declaration of Helsinki. The Japanese guidelines permit the use of data from clinical records in hospitals without consent if the data are anonymous. Hence, informed consent was not required in this study because the data were obtained anonymously from clinical records. In addition, in Japan, patients are not required to approve or disapprove the use of anonymous information from their hospital records for research. The Ethics Review Committee of the Faculty of Medicine at the University of Yamanashi waived the requirement for consent, as the research presented with no more than minimal risk and the rights and welfare of the subjects would not have been adversely affected by the waiver.

## Results

We received patient information from 110 hospitals and enrolled 75 case-control pairs in this study. Of the 150 enrolled patients, 116 were men. The mean follow-up period was 2.5 years (standard deviation [SD], 2.8). The mean ages in the case and control groups were 72.8 (SD, 7.9) and 72.7 (SD, 7.6) years, respectively. Of the 75 patients in each group, 23 patients (30.7%) in the case group were vaccinated compared with 29 patients (38.7%) in the control group. Patient data on the history of cancer, diabetes mellitus, and smoking status are listed in Table 1. Table 2 shows that exacerbation of IIP was the most frequent cause of mortality in the case group, followed by pneumonia. Table 3 lists the reasons why physicians did not vaccinate patients in either group. For the case group, the most common reason was poor physical condition. For the control group, the most common reason was a shortage of the vaccine, and the control group was cited for this reason more frequently than the case group.

**Table 1:** Patient characteristics.

Characteristic	Case	Control
**Gender, male/total**	58/75 (77.3%)	58/75 (77.3%)
**Age**	72.8 (SD 7.9)	72.7 (SD 7.6)
**Follow-up period (year)**	2.5 (SD 2.8)	2.5 (SD 2.8)
**Vaccinated for influenza A(H1N1)2009**	23/75 (30.7%)	29/75 (38.7%)
**Vaccinated for seasonal vaccine**	9/68 (13.2%)	24/70 (34.3%)
**Reported cases of influenza**	3/71 (4.2%)	0/68 (0.0%)
**History of cancer**	2/74 (31.1%)	12/72 (16.7%)
**Diabetes melitus**	35/75 (46.7%)	17/73 (23.3%)
**Current smoking**	5/75 (6.7%)	12/75 (16.0%)

SD, standard deviation.

**Table 2:** Causes of mortality.

	No. of cases
Cause of mortality	(% of total)
**Exacerbation of idiopathic interstitial pneumonia**	57 (76.0%)
**Pneumonia**	13 (17.3%)
**Cardiac dysfunction**	1 (1.3%)
**Lung cancer**	1 (1.3%)
**Myelodysplastic syndrome**	1 (1.3%)
**Unknown**	2 (2.7%)
**Total**	75 (100%)

**Table 3:** Reasons some patients did not receive the influenza A (H1N1) 2009 vaccine.

	Case patients	Control patients
Reason for non-vaccination	(% of total)	(% of total)
**Patient in poor physical condition**	16 (34.0%)	3 (7.5%)
**Shortage of new vaccine**	4 (8.5%)	11 (27.5%)
**Use of steroids**	2 (4.3%)	2 (5.0%)
**Patient rejection**	4 (8.5%)	7 (17.5%)
**Economic reason**	1 (2.1%)	0 (0.0%)
**Depression**	0 (0.0%)	1 (2.5%)
**No necessity**	0 (0.0%)	1 (2.5%)
**Unknown**	20 (42.6%)	15 (37.5%)
**Total**	47	40

The proportion of the case group that was vaccinated was lower than that of the control group, which indicates a decreased risk of mortality in vaccinated patients (crude conditional OR, 0.63; 95% CI, 0.25–1.47). Table 4 lists the ORs adjusted for diabetes mellitus, poor physical condition, and other covariates. The sample size was not large enough to determine a potential relationship between the influenza A (H1N1) 2009 vaccine and mortality rate in patients with IIP. Sensitivity analyses yielded nearly identical results as follows: (1) If an outcome was restricted to deaths due to exacerbation or pneumonia, then the crude conditional OR was 0.63 (95% CI, 0.25–1.47) and the adjusted conditional OR in Model 3 (Table 4) was 1.12 (95% CI, 0.33–4.30). (2) If case patients were restricted to exclude those in poor physical condition, then the crude conditional OR was 1.00 (95% CI, 0.35–2.84) and the adjusted conditional OR in Model 3 (Table 4) was 1.17 (95% CI, 0.30–5.09). Among the non-vaccinated patients, 23% were in poor physical condition. The simulation study (a) estimated that the genuine OR in Model 3 (Table 4) should be 0.63–0.71 (Table 5), assuming that there were the same approximate proportion of patients in poor physical condition in the vaccinated and non-vaccinated groups (0.2–0.3). The mean of conditional OR should be 0.67 and its standard error should be 0.08, in simulation (b) when the physical condition was slightly but not significantly correlated with the follow-up period in the vaccinated patients like the unvaccinated patients.

**Table 4:** Crude conditional mortality ORs^a^, adjusted conditional mortality ORs^a^, and CIs.

		Model 1	Model 2	Model 3
Variable	Crude conditional	Adjusted conditional	Adjusted conditional	Adjusted conditional
(No. of pairs considered)	OR (CI)	OR (CI)	OR (CI)	OR (CI)
**Influenza A(H1N1)2009 vaccination (75)**	0.63	0.47	1.01	1.18
	(0.25–1.47)	(0.17–1.21)	(0.38–2.72)	(0.33–4.49)
**Diabetes melitus (73)**	3.25	3.74	–	3.48
	(1.43–8.31)	(1.62–8.61)	–	(1.35–9.01)
**Poor physical condition (75)**	7.5	–	7.54	7.32
	(1.74–67.60)	–	(1.63–34.94)	(1.35–39.78)
**Seasonal influenza vaccination (67)**	0.33	–	–	0.51
	(0.08–1.10)	–	–	(0.13–2.00)
**History of cancer (71)**	2.13	–	–	1.45
	(0.87–5.69)	–	–	(0.55–3.79)
**Shortage of influenza**	0.71	–	–	1.02
**A(H1N1)2009 vaccine (75)**	(0.18–2.61)	–	–	(0.24–4.33)

OR, odds ratio; CI, 95% confidence interval.

a ORs were caluculated for case-control pairs.

**Table 5:** Results of simulation study (a) with regard to each potential probability of being in poor physical condition in vaccinated patients, 10,000 times.

Probability that the vaccinated patient is in poor physical condition	Mean estimate of conditional OR	Standard error of conditional OR estimate
0	1.18	0
0.1	0.82	0.10
0.2	0.71	0.08
0.3	0.63	0.07
0.4	0.57	0.07
0.5	0.52	0.07

OR, odds ratio.

## Discussion

To our knowledge, this is the first study to investigate the mortality rate in patients with IIP after influenza A(H1N1)2009 vaccination. All study patients were Japanese and mongoloid in origin. In our case-control study, we detected no statistically significant increased risk in mortality rate after vaccination. Our simulation study further supported no increased mortality rate from this vaccination.

Nakada et al. reported on potentially fatal adverse events in Japanese patients with underlying disease who received the influenza A (H1N1) 2009 vaccine [18]. However, those results were based on passive surveillance data and did not determine the risk of this vaccine [19]–[21]. Detection of adverse event signals is known to be difficult [22], [23]. The U.S. Vaccine Adverse Event Reporting System, which uses passive surveillance, reported 13 deaths after 46.2 million doses of influenza A (H1N1) 2009 vaccine were distributed in the United States between October 5 and November 20, 2009. Of the 13 patients who died, 9 had severe systemic disease and 1 died in a traffic accident [24]. Based on post-marketing surveillance of adverse events for 89.6 million doses of the influenza A (H1N1) 2009 vaccine administered in China, the Chinese Centers for Disease Control and Prevention reported that 10 sudden deaths occurred; 9 of these patients had cardiovascular disease, liver failure, or stroke that was followed by cerebral herniation, and the remaining patient died 43 h after vaccination and had no history of a medical condition [25]. For all 3 of these surveillance reports, the causes of deaths after vaccination were not determined.

Some randomized controlled trials (RCTs) of the safety of pandemic influenza vaccines in healthy populations have been reported. An RCT in China with 12,691 participants investigated the safety and immunogenicity of the influenza A (H1N1) 2009 vaccine and found local reactions (e.g., pain, swelling, and redness) and systemic reactions (e.g., fever, headache, and myalgia) but no instances of death [26]. A Hungarian RCT of 355 participants also investigated the safety and immunogenicity of this pandemic vaccine and did not report any deaths [27]. An Australian RCT compared responses in 240 patients after they had received 15-µg and 30-µg doses of this pandemic vaccine and found no deaths or serious adverse events [28]. Two phase 2 RCTs of adults and children in the United States reported no significant differences in solicited systemic reactions between the pandemic vaccine and placebo groups and reported no severe adverse events for either group [29]. Another RCT of the use of the live attenuated influenza A (H1N1) 2009 vaccine in healthy children and adults reported no deaths but reported 1 case of depression and 1 case of osteomyelitis in the vaccine recipients [30]. These 6 recent RCT studies may lack the statistical power required to detect slight risks of mortality of the influenza A (H1N1) 2009 vaccine; however, these studies indicate that this vaccine could be safely administered to healthy adults. A meta-analysis of the immunogenicity and tolerability of pandemic vaccines provided evidence that supports the safety of these vaccines [31].

Several recent observational studies have been conducted on the efficacy and safety of various types of pandemic influenza A (H1N1) 2009 vaccination in patients with underlying diseases or immunosuppression. Two studies of patients on hemodialysis did not report any serious adverse events [32], [33]. After the administration of pandemic vaccines to patients with HIV in 6 studies, several serious adverse events were observed, including fatigue, fever, and ecchymosis, and 1 fatal case of fulminant hepatitis was reported [34]–[39]. Of all the patients receiving anticancer treatment in 2 studies, a 70-year-old patient who had bladder cancer and multiple bone metastases died of multi-organ dysfunction 12 days after receiving the pandemic vaccine [40], [41]. Of all the patients with systemic lupus erythematosus using 1 or more immunosuppressive medications included in 2 studies, 1 reportedly experienced general malaise, sore throat, fever, and blurred vision 2 weeks after vaccination [42], [43]; no patients died. One study reported that of 390 patients with asthma aged 12 to 79 years receiving pandemic vaccines, 4 were hospitalized with asthma within 21 days of vaccination, and 1 of the 4 subsequently died [44].

In this study, the final adjusted OR of the influenza A (H1N1) 2009 vaccine (Table 5), which resulted from the simulation study (a), indicated a reversal of the adjusted OR in Model 3 (Table 4). We performed the simulation study because the surveys containing patient information on poor physical condition were compiled only to provide reasons for non-vaccination. We compensated for this information gap in vaccinated patients in both the case and control groups. After the simulation study, we assumed that the probability of poor physical condition among the vaccinated individuals was approximately identical among non-vaccinated individuals (i.e., 23%). If this assumption is correct, then the estimated adjusted conditional OR of influenza A (H1N1) 2009 vaccination to death is 0.63–0.71 (for 0.2 or 0.3 probability of poor physical condition) or less than 0.83 (for 0.1 probability of poor physical condition) in this study.

With regard to the risk of mortality of the pandemic influenza A (H1N1) 2009 vaccine in a Japanese population of patients with IIP, we developed this epidemiological study using a classic case-control approach. Due to insufficient power, previous epidemiological studies have not been able to establish determinant evidence on the safety or risk of vaccination in immunosuppressive populations, pregnant women, or children [45]–[63]. Passive surveillance studies of sufficiently high power also were unable to demonstrate the safety or risk of vaccination because the cause of adverse events in those studies could not be determined [19]–[23]. Our study suggests that the influenza A (H1N1) 2009 vaccine is of no epidemiological risk with regard to mortality rate in patients with IIP.

The strength of our study is that all cases and controls were matched for highly influential confounders, including age, gender, follow-up period, hospital, and index date. The 2 subjects in each case-control pair exhibited similar features and a nearly identical risk of death. Studies conducted previously were not designed to examine mortality [24]–[27]. After adjusting for diabetes mellitus (which sometimes emerges during the progression of IIP), we calculated a conditional OR of 0.47 (95% CI, 0.17–1.21). The physician’s explanation of “I refrained from using A (H1/N1) 2009 vaccine because my patient was in poor physical condition” for non-vaccination presents a complicated scenario; vaccinated patients were not queried on their physical condition, and mathematical proof (Appendix A) describes this caused a positive bias in the ORs that were adjusted for poor physical condition. After adjustment for diabetes mellitus, poor physical condition, seasonal influenza vaccination, history of cancer, and shortage of influenza A (H1N1) 2009 vaccine, the positively biased OR was 1.18 in Model 3 (Table 4) and was not significant. The simulation studies, which we conducted to determine the genuine conditional OR, indicated that the OR for Model 3 (Table 4) was less than 1 and presented some positive bias. These results indicated that the vaccine is of no risk for use for patients with IIP.

Our study had several limitations. Most notably, the sample size was small and provided insufficient statistical power because IIP is a rare disease, and the matching criteria were strict. However, 97.4% of case patients were matched to the eligible control patients. The enrolled subjects included both inpatients and outpatients with IIP. Although case-control studies generally serve as a powerful tool to assess rare outcomes, they are of limited use regarding rare adverse events in a small population [8], [64]. Another study limitation was “healthy user bias” [65], [66]; the vaccinated population may have contained fewer patients deemed to be in poor physical condition than the non-vaccinated population. This scenario would result in decreased ORs and occur when physicians refrain from administering a vaccine because of a patient’s poor physical condition. The extent of the severity of IIP may not have been matched or adjusted sufficiently. The clinical records for the classification of IIP should specify various subtypes, pulmonary function test results, laboratory data, and medications [10]–[12]. However, we decided that the addition of these standards to the matching factors would have reduced the sample size and power further. In addition, the lack of balance of severity was unavoidable and incidental; by definition, the case patients died within the 6-month study period. We adjusted the results to reflect the follow-up period, hospital, gender, age, history of seasonal influenza vaccination, comorbidity, poor physical condition by matching, using covariates, and adopting simulation studies [67]. These processes would have minimized the healthy user bias. The final limitation of this study was its dependence on physician reports, which were based on clinical records. However, the possibility of incorrect information about vaccination status is low because the Japanese pulmonary physicians made great efforts to describe pulmonary status, including vaccination status, especially when the supply of the pandemic vaccines was limited in the winter of 2009–2010. Due to these limitations, a potential for increased risk of mortality after vaccination has not been ruled out.

## Conclusions

We investigated the mortality rate in Japanese patients with IIP after the influenza A (H1N1) 2009 vaccination. In this study, we detected no significant increase in the mortality rate in patients with IIP who received the influenza A (H1N1) 2009 vaccine. These results, however, are limited by a small sample size and low statistical power; hence, a larger study is required.
